# Distinct immunologic changes in vivo following combination versus individual PD-1 or CTLA-4 checkpoint blockade in human cancer

**DOI:** 10.1186/2051-1426-2-S3-O7

**Published:** 2014-11-06

**Authors:** Kavita Dhodapkar, Rakesh Verma, Mario Sznol, Chandra Sekhar Boddupalli, Scott Gettinger, Harriet Kluger, Madhav Dhodapkar, Rituparna Das

**Affiliations:** 1 Yale University, New Haven, CT, USA

## 

Therapies targeting T cell immune checkpoints such as CTLA4 and PD1/PDL1 axis have shown considerable promise in the therapy of human cancer. Combination therapy with dual immune checkpoint blockade (ICB) was recently shown to be highly active in melanoma. While signaling via both PD1 and CTLA4 is known to converge downstream and dampen T cell function, data comparing in vivo effects of blockade of these immune checkpoints either alone or in combination in vivo in humans are limited. Here we have analyzed paired pre/post therapy samples from patients treated either anti-CTLA4 (n = 5) or anti-PD1 (n = 6) alone, or a combination of anti-CTLA4 and anti-PD1 (n = 8), using several methodologies including multi-parameter flow cytometry, single-cell mass-cytometry (CyTOF), Luminex and analysis of transcriptome of purified immune cells with exon-level arrays. We show that blockade of CTLA4, PD1 or combination blockade leads to distinct immunologic, genomic and cytokine signatures *in vivo*. CTLA4 blockade leads to a prominent proliferation signature in vivo, manifest as an increase in Ki-67 expression in a subset of T cells with transitional memory phenotype. PD1 blockade does not induce this phenotype and instead leads to marked changes in T cells expressing NK and cytolysis associated genes, as exemplified by Granzyme+ T cells. Combination blockade leads to non-overlapping changes in gene expression including proliferation-associated and chemokine genes and leads to an increase in both Ki67+and Granzyme+ T cells. Overall, therapy-induced changes are more prominent in T cells than in monocytes include also involve non-overlapping changes in several alternatively spliced transcripts and non-coding RNAs. Each of the ICB therapies also leads to a distinct cytokine profile with differential effects on systemic levels of sIL2R and IL1a. Changes seen in the peripheral blood T cells can also be seen in the tumor infiltrating lymphocytes. Combination therapy leads to an increase in interferon-gamma producing T cells in both circulation as well as tumor bed. PD1 expression is higher on tumor infiltrating T cells when compared to T cells in circulation. Importantly, PD1 receptor occupancy following anti-PD1 therapy may be incomplete in the tumor infiltrating T cells even in the setting of complete receptor occupancy in circulating T cells. These data demonstrate that blockade of PD1, CTLA4 alone or in combination have distinct immunologic effects *in vivo *and each strategy serves as a unique immune-therapeutic. Improved understanding of the in vivo effects of ICB is needed for rational development of future immune-based combinations against cancer.

